# The role of subchondral bone, and its histomorphology, on the dynamic viscoelasticity of cartilage, bone and osteochondral cores

**DOI:** 10.1016/j.joca.2018.12.006

**Published:** 2019-03

**Authors:** N.L.A. Fell, B.M. Lawless, S.C. Cox, M.E. Cooke, N.M. Eisenstein, D.E.T. Shepherd, D.M. Espino

**Affiliations:** † Department of Mechanical Engineering, University of Birmingham, United Kingdom; ‡ School of Chemical Engineering, University of Birmingham, United Kingdom; § Institute of Inflammation and Ageing, Queen Elizabeth Hospital Birmingham, United Kingdom; ‖ Royal Centre for Defence Medicine, Birmingham Research Park, United Kingdom

**Keywords:** Cartilage, Histomorphometry, Knee, Subchondral bone, Viscoelasticity

## Abstract

**Objective:**

Viscoelastic properties of articular cartilage have been characterised at physiological frequencies. However, studies investigating the interaction between cartilage and subchondral bone and the influence of underlying bone histomorphometry on the viscoelasticity of cartilage are lacking.

**Method:**

Dynamic Mechanical Analysis (DMA) has been used to quantify the dynamic viscoelasticity of bovine tibial plateau osteochondral cores, over a frequency sweep from 1 to 88 Hz. Specimens (approximately aged between 18 and 30 months) were neither osteoarthritic nor otherwise compromised. A maximum nominal stress of 1.7 MPa was induced. Viscoelastic properties of cores have been compared with that of its components (cartilage and bone) in terms of the elastic and viscous components of both structural stiffness and material modulus. Micro-computed tomography scans were used to quantify the histomorphological properties of the subchondral bone.

**Results:**

Opposing frequency-dependent loss stiffness, and modulus, trends were witnessed for osteochondral tissues: for cartilage it increased logarithmically (*P* < 0.05); for bone it decreased logarithmically (*P* < 0.05). The storage stiffness of osteochondral cores was logarithmically frequency-dependent (*P* < 0.05), however, the loss stiffness was typically frequency-independent (*P* > 0.05). A linear relationship between the subchondral bone plate (SBP) thickness and cartilage thickness (*P* < 0.001) was identified. Cartilage loss modulus was linearly correlated to bone mineral density (BMD) (*P* < 0.05) and bone volume (*P* < 0.05).

**Conclusion:**

The relationship between the subchondral bone histomorphometry and cartilage viscoelasticity (namely loss modulus) and thickness, have implications for the initiation and progression of osteoarthritis (OA) through an altered ability of cartilage to dissipate energy.

## Introduction

Studies concerning the pathology of osteoarthritis (OA) have, to date, mainly focused on articular cartilage and the factors that predispose cartilage to damage. Biological factors include the effects of metalloproteinase (MMPs) and aggrecanase activity, which promote cartilage degradation by affecting the cartilage matrix^1^. Physical variables affecting the progression of OA include anatomical location within the joint^2^, loading frequency^3^, ^4^, hydration^5^, and cartilage thickness^2^. It has been hypothesised that a subgroup of the population, with an impulsive^6^ heel-strike rise time in the range of 5–25 ms^7^^,^^8^, may be predisposed to developing OA through impact loading^9^. There is also the suggesting that mechanical loading of the joint initiates a biological response^10^. Articular cartilage exhibits time dependent, viscous and elastic behaviour under load. Such properties are characteristic of a viscoelastic material^11^. The frequency-dependent viscoelastic response of the articular cartilage in isolation^12^, ^13^ and in conjunction^13^, ^14^ with subchondral bone have been investigated, using Dynamic Mechanical Analysis (DMA), along with other substrates^15^.

Bone is a viscoelastic material^16^, however, DMA of bone has not been reported over a range of physiological frequencies. Investigation of the viscoelastic behaviour of bone at physiological frequencies is important since outside of this range the frequency response is unrepresentative of the *in vivo* mechanics of cartilage^4^. It has been hypothesized that the health of cartilage depends on the mechanical properties of this subchondral bone^17^. Viewed as a whole joint disease, current research into the pathogenesis of OA is focused on the cartilage-bone unit^18^ and is demonstrating that the subchondral bone in OA is important in both treatment methods^19^ and understanding disease initiation^20^. The interaction between cartilage and bone, and the potential influence of histomorphological properties, as an indicator of bone health, on the viscoelastic behaviour of bone (and potentially cartilage), has not been assessed. The clinical significance of a correlation between these properties manifests in the ease of obtaining histomorphological properties of bone by utilising dual-energy X-ray absorptiometry (DEXA) and computed tomography (CT) scanning.

As OA is a disease of both the bone and cartilage, it is important to assess their interaction as a functional unit^10^, ^21^ and also the mechanical properties of each tissue individually. The primary aim of this study was to characterise the relationship between the cartilage and bone through analysis of the viscoelastic properties both as a combined structure (osteochondral core) and as separate entities. The secondary aim was to use micro-CT (μCT) scans of the subchondral bone to identify whether a relationship exists between the histomorphometric parameters and the viscoelastic properties of the cartilage and bone.

## Methods

### Specimen preparation

Six skeletally mature bovine knee joints (approximately aged between 18 and 30 months) were obtained from Dissect Supplies UK (Birmingham, UK), following standard practices^2^, ^12^, ^14^, ^22^. Upon arrival in the laboratory, the tibiae were wrapped in tissue paper saturated in Ringer's Solution and stored in the freezer at −40°C until required for testing^2^, ^5^, ^12^, ^14^, ^22^. Previous studies have shown that freezing does not change the mechanical properties of articular cartilage^23^. Medial and lateral regions were isolated along the central eminence using a hand saw. Two specimens (covered and uncovered by the meniscus) were obtained from both the medial and lateral regions, giving a total of 24 specimens.

Prior to testing, a specimen was isolated and thawed for 12 h at room temperature. India ink (Loxley Art Materials, Sheffield, UK) was used to indicate any damage to the articular surface^24^; specimens were neither osteoarthritic nor otherwise compromised^2^, ^4^, ^5^, ^23^. The area covered and uncovered by the meniscus were marked and separated. Further dissection, using a hand saw, isolated cartilage-bone cubes (*n* = 24) with a volume of approximately 14 × 14 × 14 mm^14^. The size of the cube was determined during preliminary investigations and was optimised for adequate X-ray transmission during μCT scans, whilst still providing sufficient area for viable drilling.

### Micro computed tomography (μCT)

Cartilage-bone cubes were scanned using a SkyScan 1172 micro-CT system (Bruker, Belgium). Preliminary analysis identified the optimal settings for μCT, based on transmissibility and trabeculae size. Individual specimens were scanned with 80 kV voltage, 8 W beam power, aluminium and copper filer, exposure time of 2150 ms, and 12.03 μm pixel size (1740.99 μm^3^ voxel size). The specimen positioning and orientation was optimised according to Kumar *et al.*^25^. Data were reconstructed using NRecon software (Version 1.6.10.2, Bruker); all specimens used the same reconstruction parameters. A beam hardening of 20%, smoothing and ring artefact reduction factor of 2 and upper and lower attenuation coefficient of 0.03 and 0.00, respectively, was used for reconstruction. The histomorphological analysis was conducted using CTAn software (version 1.15.4.0, Bruker) following the Bone Mineral Density (BMD) and Tissue Mineral Density (TMD) Method Note^26^ where the models were calibrated using hydroxyapatite (HA) phantom rods of known mineral concentration with densities of 250 and 750 mg cm^−3^. The phantom rods were scanned and reconstructed with the same parameters as the samples as a baseline in CTAn for correlating greyscale and density. The subchondral bone layer was identified visually. Due to a scanning error with five of the specimens, only 19 specimens were used in histomorphological analysis.

## Viscoelastic testing

Following scanning, a pillar drill, with a 10 mm outer diameter core drill bit (8 mm inner diameter), was used to isolate osteochondral cores^15^. The 8 mm diameter specimens were cut down to a bone depth of 3 mm and hydrated in Ringer's Solution for 30 minutes^27^. The cores were tested using the DMA protocol outlined below. Following this test, the cartilage was isolated from the bone, using a scalpel blade, and the tissues were hydrated for 30 minutes before testing isolated cartilage and bone tissue using the same DMA procedure.

The viscoelastic properties of the osteochondral cores, articular cartilage and subchondral bone were determined using a Bose ElectroForce 3200 and corresponding WinTest DMA software (Bose Corporation, Eden Prairie, MN). This method has been used previously in analysing the viscoelastic response of articular cartilage in isolation^12^, ^13^ and in conjunction^13^, ^14^ with subchondral bone. A sinusoidally varying compressive load, between 37.7 N and 85.5 N, was applied to each specimen under unconfined conditions; this range was used to induce a maximum nominal stress of 1.7 MPa, which approximates physiological loading in the lower limb^28^, and has been used in various studies^2^, ^5^, ^12^, ^13^, ^14^, ^22^. A cylindrical stainless steel compression plate, with a diameter of 20 mm, applied the force consistently over the full specimen surface (with samples resting on an aluminium base plate). All specimens were tested at room temperature, consistent with previous studies^2^, ^4^, ^5^, ^23^. Dehydration over the short duration of the test has been shown to be insignificant^12^, ^13^.

For measurement of storage and loss properties, a dynamic “steady-state” sweep was necessary prior to the testing frequencies. For cartilage, this occurs between 1200 and 4500 cycles^14^, ^29^. This was achieved by subjecting the specimens to two preload conditions of 25 Hz for 1500 cycles and 50 Hz for 3000 cycles with a 60 s rest period between frequencies^2^, ^12^, ^13^, ^14^, ^22^. After pre-cycling, the sinusoidal force range was applied at eight different frequencies (1, 8, 10, 12, 29, 49, 71, 88 Hz). The frequency range covered the physiological frequencies associated with healthy gait (1 Hz with a heel strike rise time of 500 ms) to rapid heel strike rise times (88 Hz with a rise time of 5.68 ms) which is seen in a sub-group of the population that may be predisposed to developing OA through impact loading^9^.

At each frequency, the DMA software performed a Fast Fourier Transform (FFT) of the force and displacement sinusoids and the FFT provided the magnitude of displacement (d*), the magnitude of load (F*), the phase lag (*δ*) and the actual frequency (*f*)^30^. Next, the software calculated the complex (k*), storage (*k’*) and loss (*k’’*) stiffness (Eqs. (1), (2), (3), respectively)^30^. A cylindrical shape factor (Eq. (4)) was calculated for each specimen, dependent on diameter (*D*) and height (*h*). The dimensions of the bone were measured using a Vernier Calliper. The cartilage thickness used for calculating viscoelastic properties was measured using a needle indentation method^2^, ^14^. The shape factor (*S*; Eq. (4)) was used to calculate the storage and loss modulus (*E*′ and *E″*, respectively) of cartilage (Eqs. (5), (6)))^14^.

(1) $$k^{*}=\frac{F^{*}}{d^{*}}$$

(2) $$k’=k^{*}cos\text{δ}$$

(3) $$k''=k^{*}\text{sinδ}$$

(4) $$S=\frac{\pi D^{2}}{4h}$$

(5) $$E^{'}=\frac{k^{'}}{S}$$

(6) $$E''=\frac{k^{''}}{S}$$

### Quasi-static testing

A load frame instrument (Bose ElectroForce 3300, Bose Corporation, Eden Prairie, MN) was used to perform a compression test to calculate the Young's Modulus for each bone specimen. Each specimen was compressed at a displacement rate of 0.08 mm/s to failure, or to 66% strain. This compression was performed following the DMA test described previously.

### Statistics

All statistical analyses were performed using SigmaPlot 13.0 (SYSTAT, San Joes, CA, USA). The 95% confidence intervals were calculated. Regression analyses evaluated the significance of the linear fit for a combination of different variables measured, including viscoelasticity with frequency and histomorphometry of bone. Results with *P* < 0.05 were considered significant. Wilcoxon Rank–Sum tests were used to identify significant differences (*P* < 0.05) between the properties of the osteochondral core and its components (*n* = 24).

## Results

### Dynamic viscoelasticity

**Fig. 1:**
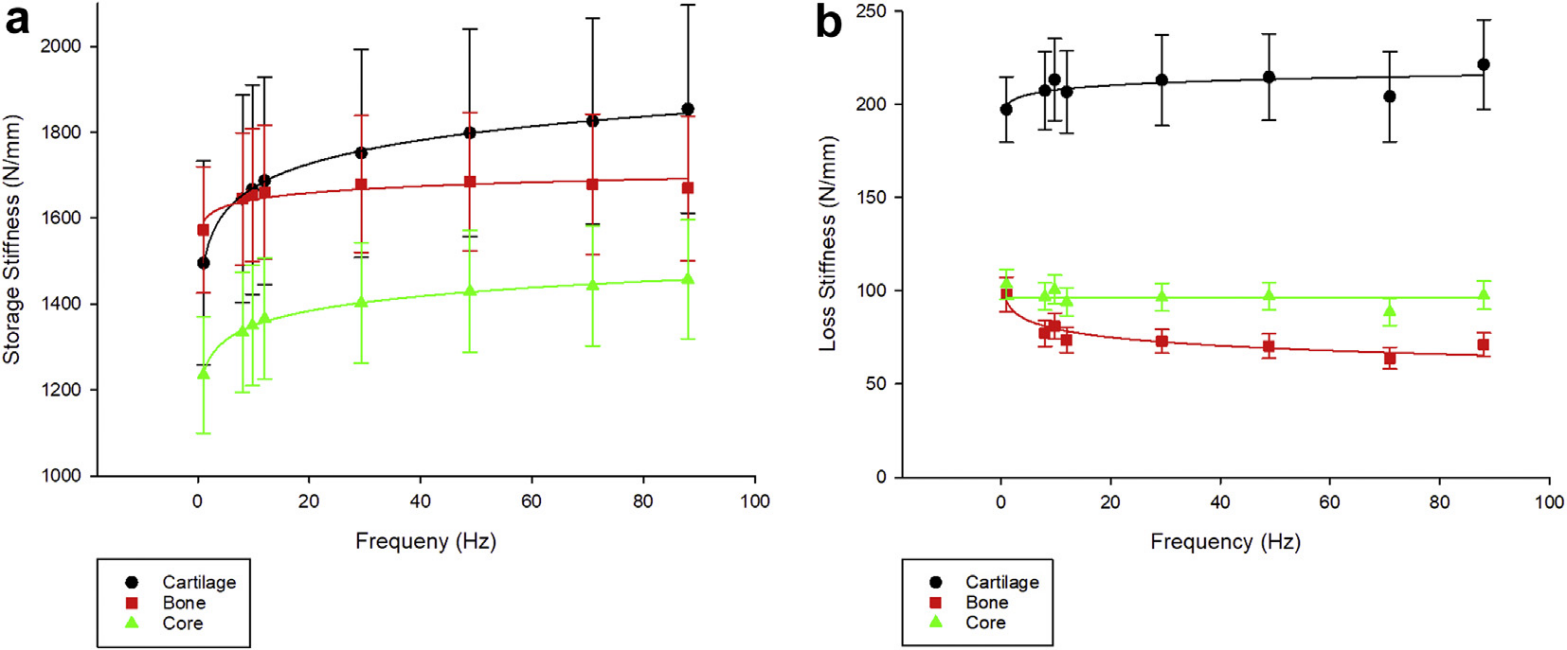
(a) Storage (*kʹ*) and (b) loss (*kʺ*) stiffness against frequency (Hz) for cartilage, bone and osteochondral core specimens (mean ± 95% confidence intervals; *n* = 24).

The cartilage, bone and osteochondral cores were viscoelastic across the frequency range tested (1–88 Hz; Fig. 1). For all frequencies, the storage stiffness was greater than the loss stiffness for each tissue. The storage stiffness of the cartilage followed a significant logarithmic trend with respect to frequency (*P* < 0.001, Eq. (7), Supplementary Information 1). Most cartilage specimens followed the same trend for loss stiffness with 17 of the 24 specimens proving significant (*P* < 0.05, Eq. (8), Supplementary Information 2).

(7) $$k^{'}=Aln\left(f\right)+B\;for\;1\;\leq f\leq 88\;Hz$$

(8) $$k{'}^{'}=A_{L}ln\left(f\right)+B_{L}\;for\;1\;\leq f\leq 88\;Hz$$

The bone storage and loss stiffness relationship with frequency were defined by a logarithmic fit (Eqs. (7), (8)), respectively). For the storage stiffness, eight of the 24 specimens did not follow the logarithmic trend (*P* > 0.05, Supplementary Information 1). The loss stiffness of bone decreased with frequency, giving the coefficient *A*_*L*_ a negative value (Eq. (8), Supplementary Information 2). Of the 24 specimens, 22 followed this trend (*P* < 0.05, Supplementary Information 2).

The storage stiffness of cores followed a significant logarithmic trend (Eq. (7), Supplementary Information 1) with respect to frequency, whereas the loss stiffness was categorised as frequency independent (Fig. 1, *P* > 0.05). Most specimens (16 of 24) followed this trend (*P* > 0.05, Supplementary Information 2).

**Fig. 2:**
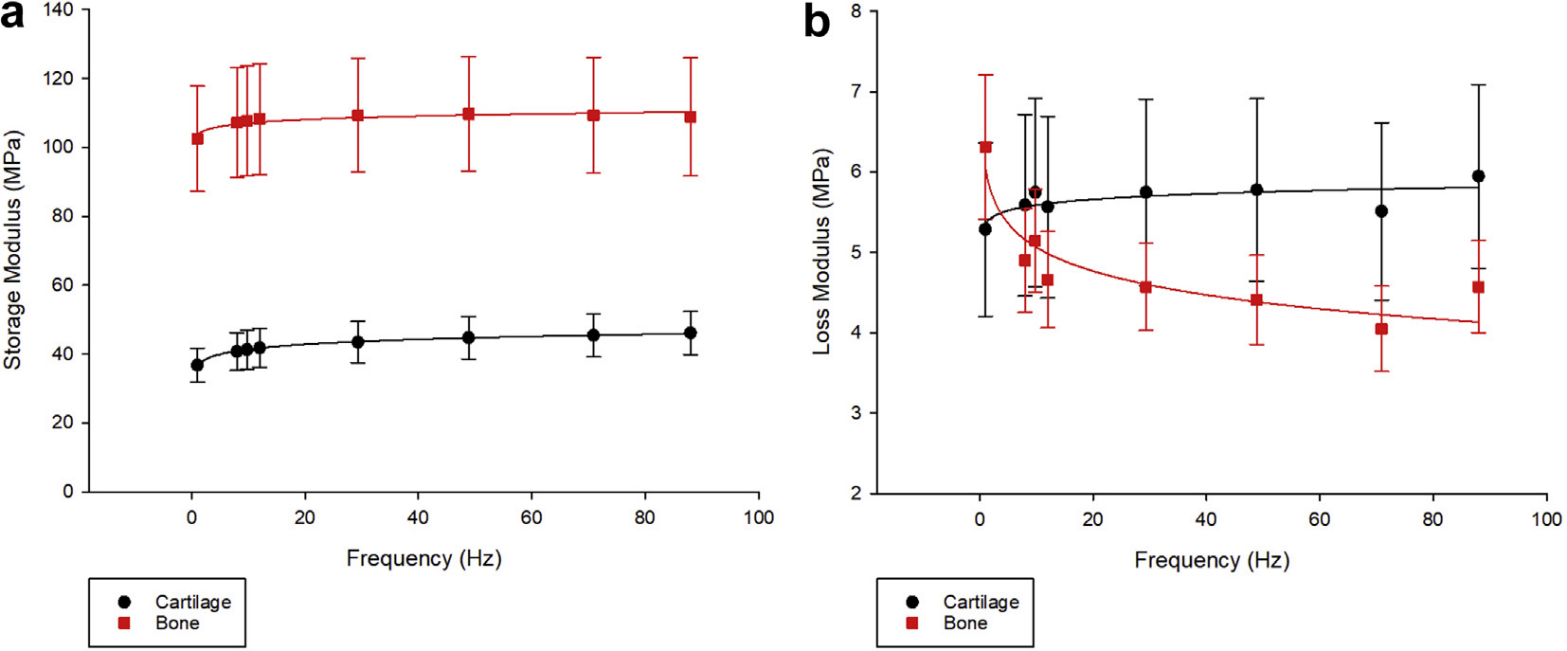
(a) Storage (*E′*) and (b) loss modulus (*E″*) against frequency (Hz) for cartilage, bone and osteochondral core specimens (mean ± 95% confidence intervals; *n* = 24).

The storage modulus of the cartilage (24 of 24) and bone (16 of 24) specimens had logarithmic relationships with respect to frequency (Eq. (9); *P* < 0.05, Supplementary Information 3). The loss modulus of cartilage and bone also followed a logarithmic trend with increasing frequency (Eq. (10)), *E″* increases for cartilage and decreases for bone (Fig. 2). Of the 24 specimens, 18 cartilage specimens and 22 bone specimens followed these trends significantly (*P* < 0.05). Regression analyses can be found in Supplementary Information 3.

(9) $$E^{'}=Cln\left(f\right)+D\;for\;1\;\leq f\leq 88\;Hz$$

(10) $$E{'}^{'}=C_{L}ln\left(f\right)+D_{L}\;for\;1\;\leq f\leq 88\;Hz$$

### Histomorphological analysis

**Fig. 3:**
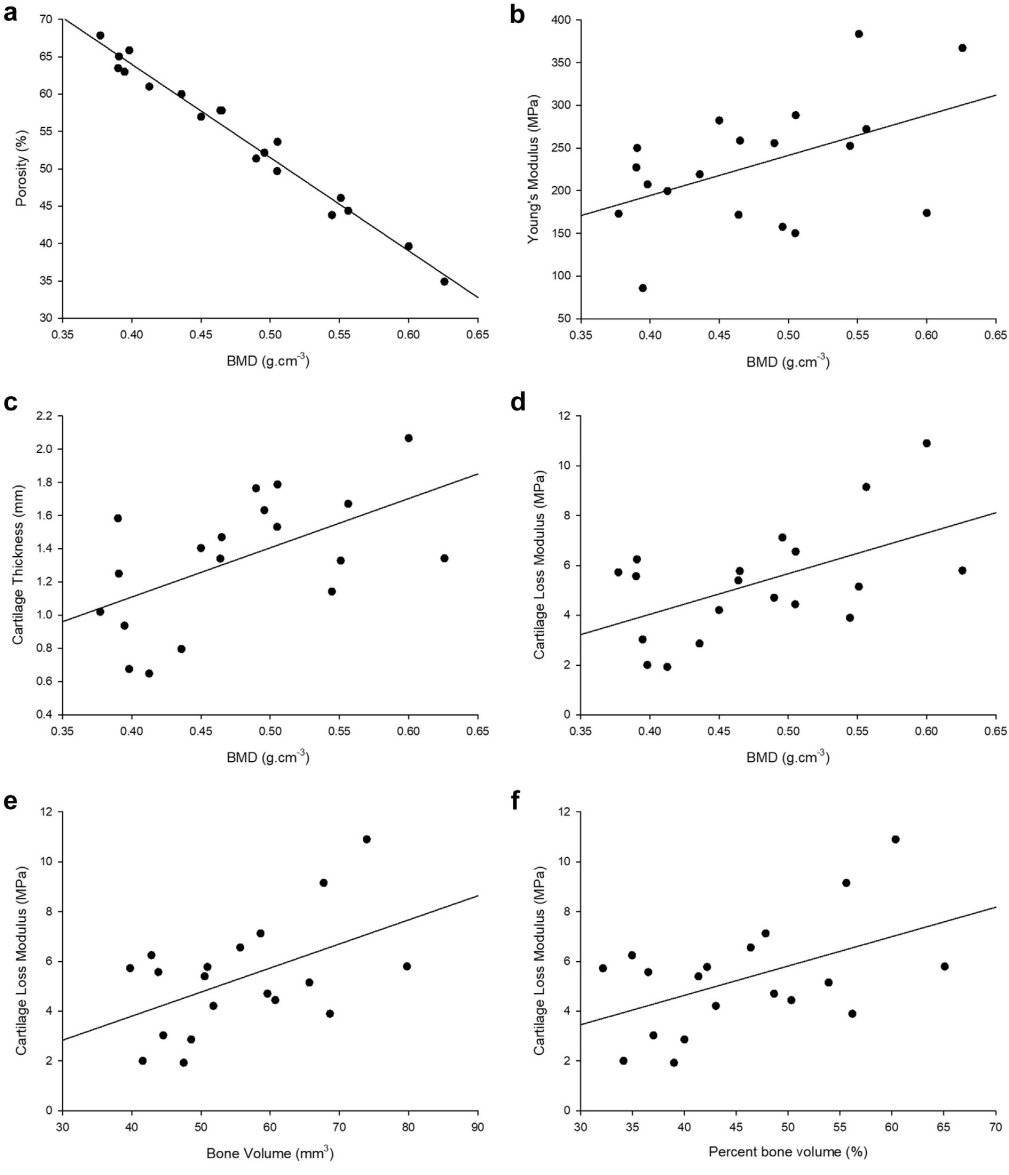
Plots of (a) Porosity (%) vs bone mineral density (BMD) (g cm^−3^), (b) Young's modulus (MPa) vs BMD (g cm^−3^) and (c) cartilage thickness (mm) vs BMD (g cm^−3^). Cartilage loss modulus, at 1 Hz, vs (d) BMD (g cm^−3^), (e) bone volume (mm^3^) and percentage of bone volume (BV/TV) (%). Regression trendline is visible in all plots (note, *n* = 19 for all plots).

Regression analysis of BMD of the respective subchondral bone (*ρ*) and percentage porosity (*Po*_*tot*_) of the bone specimens showed a significant linear relationship [Fig. 3(a)**;** *r*^2^ = 0.977; *P* < 0.001]. BMD with Young's Modulus *(E)* of bone [Fig. 3(b)] and *ρ* with cartilage thickness [Fig. 3(c)] displayed significant linear relationships (*P* < 0.05); the coefficients and constants of these equations and r^2^ values can be seen in Table I. The trends follow Eq. (11) where x represents the various properties (cartilage thickness [mm], *Po*_*tot*_ [%] and *E* [MPa]) detailed in Table I.

(11) x=F(ρ)+G

**Table I:** Linear regression analyses coefficients (*F*), constants (*G*) and significance of BMD (g/cm^3^) with cartilage thickness (mm), percentage porosity (%) and Young's modulus (MPa) (see Eq. (11))

x	F	G	*r*^2^	*P*
Cartilage thickness (mm)	3.0 mm/g cm^−3^	−0.08 mm	0.31	0.011
Porosity (%)	−124.8 %/g cm^−3^	113.9 %	0.98	<0.001
Young's Modulus (MPa)	470.6 MPa/g cm^−3^	6.1 MPa	0.23	0.037

The relationship between the loss modulus of the cartilage (*E″*_*Cart*_*)* and BMD [Fig. 3(d)], bone volume [Fig. 3(e)], and percentage bone volume [Fig. 3(f)] were investigated. *E″*_*Cart*_ was found to be significantly correlated to the BMD (*ρ*) of the respective subchondral bone (Eq. (12)), at all frequencies tested (*P* < 0.05). Significant linear relationships were found for bone volume (*Bv*) with the loss modulus of cartilage (*E″*_*Cart*_*)* for all frequencies (Table II), supporting the finding with BMD. Further, significant linear relationships were found between percentage bone volume (*Bv/Tv*) and *E″*_*Cart*_ for all frequencies except for 30 Hz (*P* = 0.051, Table II). The trends follow Eq. (12) where *α* represents the various properties (*BMD* [g.cm^−3^], *Bv* [μm^3^] and *Bv/Tv* [%]) and the coefficients *H* and *J* for each frequency are detailed in Table II **(**to avoid repetition).

(12) $$E'{'}_{Cart}=H\left(\alpha \right)+\;J$$

**Table II:** Linear regression analyses of coefficients (*H*), constants (*J*) and significance cartilage loss modulus (MPa) with respect to bone mineral density (*BMD*), bone volume (*Bv*) and percentage bone volume (*Bv/Tv*), at each frequency tested (see Eq. (12))

Freq	BMD				BV				BV/TV			
	H	J	*r*^2^	*P*	H	J	*r*^2^	*P*	H	J	*r*^2^	*P*
1	16.3	−2.5	0.30	0.016	0.097	−0.066	0.252	0.029	0.12	−0.08	0.25	0.030
8	16.7	−2.4	0.28	0.019	0.099	0.113	0.241	0.033	0.12	0.10	0.24	0.035
10	17.2	−2.4	0.28	0.020	0.102	0.121	0.237	0.034	0.12	0.11	0.23	0.036
12	16.4	−2.2	0.27	0.022	0.097	0.207	0.231	0.037	0.12	0.20	0.23	0.040
29	16.1	−1.9	0.25	0.030	0.095	0.489	0.210	0.049	0.12	0.48	0.21	0.051
49	16.0	−1.8	0.26	0.027	0.095	0.542	0.218	0.044	0.12	0.53	0.21	0.046
71	15.2	−1.7	0.25	0.031	0.091	0.461	0.215	0.046	0.11	0.45	0.21	0.048
88	15.9	−1.6	0.25	0.029	0.096	0.643	0.221	0.042	0.12	0.63	0.22	0.045

No significant relationships existed between the storage moduli of the cartilage and bone or with the loss modulus of bone. Young's modulus (*E*) of bone was also found to vary linearly with *Bv*, *Bv/Tv* and *Po*_*tot*_ (Table III; Eq. (13)). The term y characterises various properties (*Bv* [μm^3^], *Bv/Tv* [%] and *Po*_*tot*_ [%]) detailed in Table III (to avoid repetition).

(13) y=L(E)+M

**Table III:** Linear regression analyses coefficients (*L*), constants (*M*) and significance of Young's modulus (MPa) with histomorphological properties (see Eq. (13))

y	L	M	*r*^2^	*p*
**Bv** (μm^3^)	72.42 × 10^6^ μm^3^/MPa	38.69 × 10^9^ μm^3^	0.21	0.050
**Bv/Tv** (%)	0.06 %/MPa	31.39 %	0.21	0.048
**Po**_**tot**_ (%)	−0.06 %/MPa	67.98 %	0.21	0.050

**Fig. 4:**
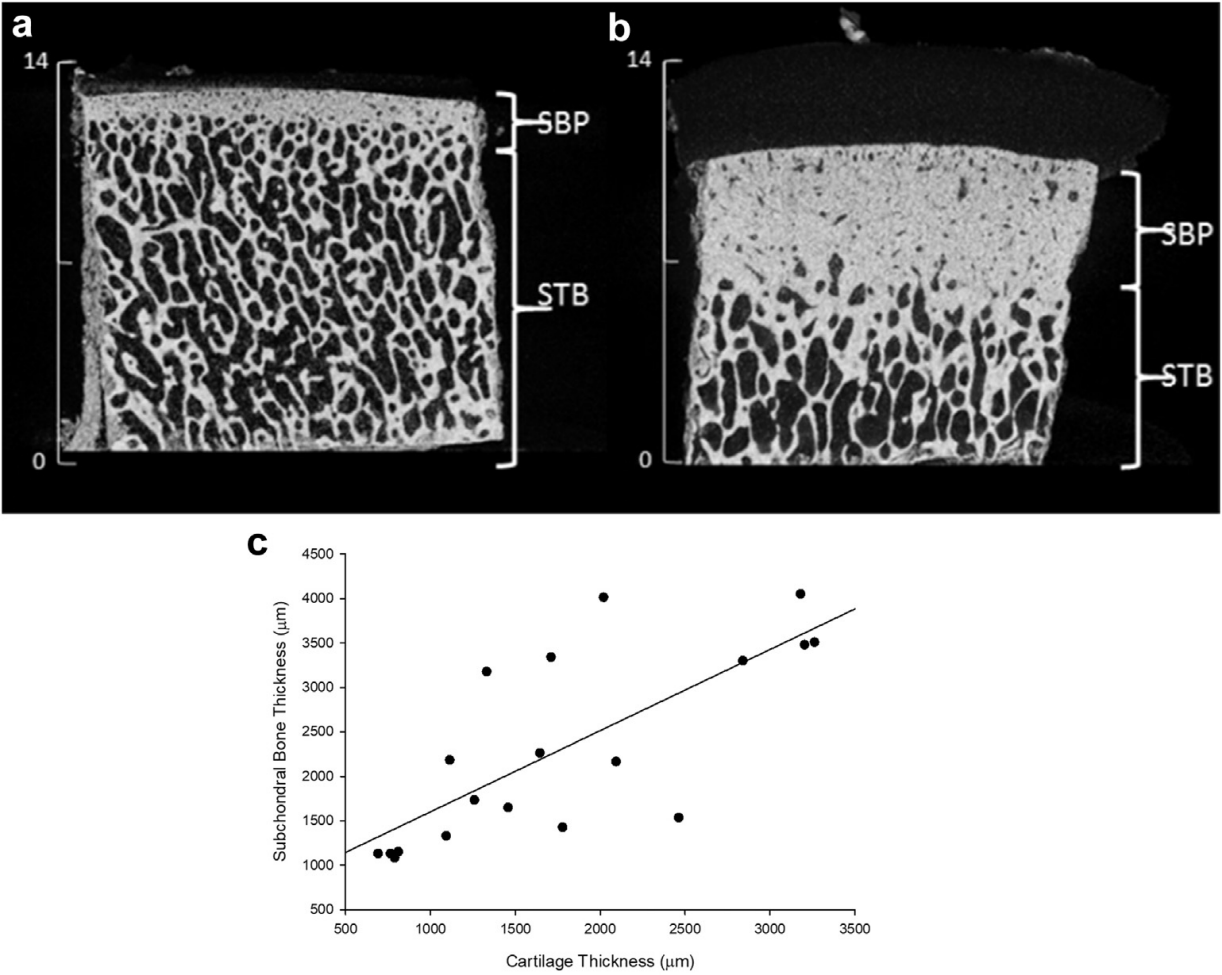
Micro-CT cross sectional image of scanned specimen taken from (a) covered and (b) uncovered lateral regions of the tibial plateau. Subchondral bone regions identified as subchondral bone plate (SBP) and subchondral trabecular bone (STB) with scale in millimetres. (c) Plot of subchondral bone plate thickness (μm) vs cartilage thickness (μm) measured from reconstructed μCT scans (note, *n* = 24).

Measurements of subchondral bone plate (SBP) thickness (*t*_*SBP*_) and cartilage thickness (*t*_*cart*_), evaluated using the reconstructed μCT images [Fig. 4(a) and (b)], were compared [Fig. 4(c)] and a significant linear relationship (Eq. (14); *r*^2^ = 0.56, *P* < 0.001), was identified. SBP thickness of two anatomically different specimens from the same joint (covered and not covered by the meniscus) is represented in Fig. 4. It was observed that specimens not covered by the meniscus *in situ* had larger SBP thickness compared to covered. The overlying cartilage thickness measurements for each specimen [covered and uncovered, Fig. 4(a) and (b)] were 0.94 and 1.67 mm, respectively.

(14) $$t_{SBP}=0.91\;t_{cart}+0.69$$

## Discussion

This study measured the viscoelasticity of osteochondral cores and the individual tissues in isolation. The subchondral bone was demonstrated to be viscoelastic and frequency dependent. This is the first study to demonstrate a correlation between histomorphological characteristics of underlying bone and the viscoelasticity of the cartilage specimens. The potential causal changes in subchondral bone and cartilage imply a dynamic relationship, not yet been quantified.

### Viscoelasticity and bone-cartilage interaction

The frequency-dependent viscoelastic response of the articular cartilage in this study is consistent with previous works^5^, ^12^, ^13^, ^14^. Further, the storage stiffness and modulus of bone increased logarithmically with frequency. The loss stiffness and modulus of the bone decreased with frequency. As such, the viscoelasticity of bone is frequency dependent. DMA results of bone are limited as the majority of studies on its viscoelasticity are taken using other methods, such as indentation^31^. One account of DMA on bone was found where cortical bone from human femora was tested at 1 Hz with a dynamic stress of 2.1 MPa and analysed based on only phase difference and storage modulus^16^. In this study, storage and loss modulus of bone were both proportional to frequency, performing DMA at a single frequency overlooks this dependency.

The storage stiffness of the osteochondral cores were found to increase logarithmically with increasing frequency, consistent with previous studies^2^, ^4^, ^5^, ^13^, ^14^. Previous studies found the dissipation property of cartilage to be frequency-independent in the form of the osteochondral core and frequency-dependent in isolation^12^, ^13^; consistent with this present study. The effect of the interaction between the cartilage and underlying bone on the viscoelastic response highlights the importance of testing the cartilage and bone as a functional unit, as opposed to separate tissues, if an understanding of an *in situ* response is required^15^.

The interaction between subchondral bone and articular cartilage has been analysed previously with the suggestion that the underlying bone restricts the cartilage from excessive lateral deformation during axial loading, therefore, affecting its apparent mechanical properties^2^, ^4^, ^11^, ^32^. This idea has been used to explain the change in frequency dependency of cartilage on and off bone^2^, ^4^, ^32^. However, the trends of the loss moduli of the isolated tissues, in this present study, suggest a cancelation in loss stiffness (and modulus), as *kʺ*_*bone*_ decreases and *kʺ*_*cart*_ increases with frequency. The opposing trends might be causal to aid joint homeostasis and damage prevention. Further, the loss modulus of cartilage in isolation increased with frequency, implying more energy is dissipated and in the case of the osteochondral core, to the bone. As bone has a higher capacity to remodel and repair than cartilage^33^, it may be an adaptive response for damage to occur in the bone preferentially; preventing long-term loss of joint function.

Through aging, bone resorption surpasses formation and consequently a decrease in BMD causes a decrease in mechanical strength^34^. Therefore, under the same load through cartilage, the bone may not be able to respond physiologically, causing micro-fracture within the subchondral bone which potentiates the progression of OA^35^. In support of this, Radin *et al.*^36^ also reported that fatigue failure of bone under repetitive impact loading is a factor in the aetiology and progression of OA. Further, higher frequency impact loading is associated with cartilage damage and has implications for the progression of OA^22^. At high frequencies, the *k’/k’’* ratio of the core increases implying more energy is stored than dissipated, increasing the likelihood of cracks in the cartilage^4^, ^13^. The storage to loss ratio of bone also increases with frequency, due to the decreasing loss stiffness, implying that either the energy is dissipated in the form of bone fractures or is transferred back to the cartilage which may become damaged at high frequencies^22^.

### Histomorphometry and the bone-cartilage unit

Many studies have postulated that repetitive impact loading caused by increased heel strike rise times, will impact the subchondral bone properties leading to an increased rate of OA progression^3^, ^4^, ^15^, ^29^, ^17^. Typical effects of OA include thickening of the subchondral plate, and an increased stiffness and thinning of the articular cartilage, causing pain and reduced mobility^20^, ^35^. The instigator of initialisation of OA, however, is yet to be determined and there may be causal effects between the tissues^21^, ^19^. Regression analysis of BMD, of the underlying bone, with cartilage *E″* identified a significant relationship at each frequency tested; a finding not previously known. Further, *Bv* and *Bv/Tv,* highlighted significant linear relationships at specific frequencies, with cartilage *E’’*. Studies have previously correlated the histomorphological properties of bone with mechanical characteristics including Young's modulus^37^, however, no studies have examined the potential relationships with viscoelastic properties of cartilage and bone. An increase in loss modulus of cartilage could imply that more of the energy transferred to the cartilage is dissipated to the subchondral bone. Repetitive loading of bone can stimulate bone remodelling, therefore, increasing BMD^38^. Alternatively, a change in BMD could change the load transmitted through the cartilage, to the underlying bone, affecting the cartilage response. Radin and Rose^17^ postulated that the health of the overlying cartilage is dependent on the mechanical properties of bone; a finding supported by this study. Radin and Rose^17^ also hypothesised that an increase in BMD would increase stiffness, reducing the viscoelastic properties of bone; however, this hypothesis remains unsupported as no significant relationship was identified in this present study.

The two distinct anatomic entities identified in the subchondral bone (SBP and subchondral trabecular bone (STB))^20^, varied between anatomically different specimens (lateral covered and lateral uncovered). A variation in SBP thickness, has been observed in the tibial plateau of ovine specimens^39^. The regression analysis with cartilage thickness suggests that SBP thickness is correlated, or is affected by the overlying cartilage, as proposed elsewhere^17^. BMD also correlated with cartilage thickness. Previously, cartilage thickness in bovine tibial specimens has been related to the strength of subchondral bone^40^. As cartilage thickness is linked to viscoelasticity and OA progression^2^, a relationship between cartilage and subchondral bone properties could be useful for diagnosis. The relationships of cartilage thickness with SBP thickness and loss modulus support the link found between cartilage loss modulus and BMD. The postulation that the health of the overlying cartilage is dependent on the mechanical properties of the subchondral bone^17^ is further reinforced by the observation that BMD, *Bv* and *Bv/Tv* were proportional to the loss modulus of cartilage.

The significant negative correlation identified between BMD and porosity (*r*^2^ = 0.977, *P <* 0.001), was consistent with a previous study of human femoral diaphysis (*R* = 0.83, *P <* 0.0001, *n* = 24)^41^. Despite the difference in species, the correlations agree with each other. Studies have concluded that BMD and porosity were good predictors of mechanical strength of cortical bone^41^, ^42^. Wachter *et al.*^41^ also identified a significant relationship between BMD and elastic modulus (*R* = −0.67, *P* < 0.05); a relationship also presented in this (*r*^2^ = 0.23, *P* = 0.037) and other studies^43^. *Bv* and *Bv/Tv* are good predictors of bone failure (for human vertebrae)^44^ and in this study were correlated to the Young's modulus of samples.

The combination of the viscous response of the cartilage and bone, together with the regression between *E″*_*Cart*_ and BMD, and the impact of loading on the SBP thickness, raise the potential for a symbiotic relationship between cartilage and bone. The histomorphological properties may contribute to energy dissipation of the osteochondral core and mechanical properties of bone. The correlation of *Bv* and *Bv/Tv* with loss moduli of cartilage further supports this idea. The structural alterations to bone and cartilage, which appear interlinked, have implications on the pathology of OA. The meniscus uncovered region of tibial plateau cartilage has been suggested as being representative of the early onset of OA^2^, which in this study matched a thicker subchondral bone with implications for cartilage thickness. Thus, structural changes during the early onset of OA may depict mutual causality. There is future scope for the identified relationship of cartilage mechanical properties to bone histomorphometry to be used in analysing the severity or progression of the disease. This would require the development of measuring bone mechanical properties (as related to density) through CT scans of bone and extrapolation of data to assess the cartilage.

### Limitations and future work

A limitation of this study is the use of bovine specimens as opposed to human tissue. However, bovine and human cartilage follow the trends for dynamic viscoelasticity despite bovine being approximately two times stiffer than human^12^. The viscoelasticity recorded is believed to be intrinsic because timescales of testing are orders of magnitude below those over which meaningful poro-elastic effects might take place, however, further studies would be required to confirm this. Viscoelastic properties vary across the tibial plateau and can be linked to distinct loading history^2^. In this study four samples were obtained from each joint from: meniscus covered, meniscus uncovered from the medial and lateral tibial plateau. This approach may raise questions around limitations due to repeated measurements. However, averaging viscoelastic and geometric data from these four regions can be misleading as bone and cartilage from these four regions have clear differences in loading history. Thus, no correction has been used for repeated measures. The findings in this study support further regional analysis, not presented in this study in part because of the number of knee joints used (*n* = 6) and the limitations which this presents. Indeed it is the very much the variability across the knee which guided the focus of this study on the correlation between material properties of cartilage and bone cores with BMD. Although the goodness of fit is low between loss modulus of cartilage and BMD, they are comparable to *r* and *r*^*2*^ values presented in BMD studies^41^, ^43^. Critically, a relationship between the ability of articular cartilage to dissipate energy and the BMD of bone appears to be significant. For the BMD measurements in this study, a constant volume was used for each specimen and no significant difference between regions was observed. The time between sample thaw completion and testing completion (μCT and DMA) was approximately 4 hours, although this includes rehydration in Ringer's solution, it is considerable. While no changes to conclusions are anticipated^23^, bias may have been introduced.

## Conclusions

The novel findings of this study are as follows:

• There is a linear relationship between the cartilage loss modulus and the bone mineral density (BMD) of the subchondral bone.
• There is a linear relationship between the cartilage loss modulus and histomorphological parameters.
• The viscoelasticity of bone was frequency dependent over a physiological frequency range.
• The frequency-dependency of cartilage loss stiffness changes in isolation or *in situ* (osteochondral core).
• A linear relationship exists between cartilage thickness and subchondral bone plate (SBP) thickness.

The combination of findings suggests a possible symbiotic, dynamic relationship between cartilage and bone.

## Author contributions

All authors were involved in the design of this study. NLAF prepared the specimens. NLAF, SCC, MEC and NME were involved in the acquisition, analysis and interpretation of the histomorphological data. NLAF, BML, DETS and DME were involved in the acquisition, analysis and interpretation of the viscoelastic and quasi-static data. During drafting of this article, all authors were involved in the critical revision. All authors approved the final, submitted version. NLAF, BML, SCC and DME take responsibility for the integrity of the work as a whole, from inception to finished article.

## Competing interests

No authors have identified any competing interests.

## Role of funding source

The equipment used in this study was funded by Arthritis Research UK (Grant number H0671). The micro-CT used in this study was funded through the Birmingham Science City Advanced Materials Project 1: Creating and Characterising Next Generation Materials Project, with support from Advantage West Midlands (AWM) and partial funding from the European Regional Development Fund (ERDF). Funding bodies have not been involved in drafting the manuscript nor data analysis.
