# Supplementary material for: The role of subchondral bone, and its histomorphology, on the dynamic viscoelasticity of cartilage, bone and osteochondral cores
Source: Osteoarthritis Cartilage. 2019 Mar;27(3):535–43. doi: 10.1016/j.joca.2018.12.006 (PMC6414396; doi:10.1016/j.joca.2018.12.006)
Supplement: Multimedia component 1 [file mmc1.docx]

**Supplementary Information 1**: Storage stiffness (Eq. 7) regression analyses of the osteochondral core and its constituents. Stiffness coefficients (A) and constants (B) are in N/mm. p < 0.05 indicates that the logarithmic regression analysis was significant.

|  | **Cartilage k’** | | | | **Bone k’** | | | | **Core k’** | | | |
| --- | --- | --- | --- | --- | --- | --- | --- | --- | --- | --- | --- | --- |
|  | **A** | **B** | **r^2^** | **p** | **A** | **B** | **r^2^** | **p** | **A** | **B** | **r^2^** | **p** |
| **1** | 120.6 | 1475 | 0.982 | <0.001 | 27.14 | 1822 | 0.892 | <0.001 | 45.61 | 1488 | 0.982 | <0.001 |
| **2** | 106.4 | 1532 | 0.999 | <0.001 | 11.04 | 1493 | 0.197 | 0.27 | 52.52 | 955.9 | 0.998 | <0.001 |
| **3** | 88.69 | 1758 | 0.986 | <0.001 | 19.30 | 1472 | 0.907 | <0.001 | 36.78 | 1634 | 0.915 | <0.001 |
| **4** | 90.71 | 1255 | 0.990 | <0.001 | 32.83 | 1680 | 0.927 | <0.001 | 43.81 | 794.2 | 0.994 | <0.001 |
| **5** | 83.96 | 1981 | 0.992 | <0.001 | 15.79 | 1554 | 0.408 | 0.088 | 54.41 | 1558 | 0.990 | <0.001 |
| **6** | 67.65 | 1068 | 0.997 | <0.001 | 20.85 | 1514 | 0.819 | 0.002 | 62.21 | 1042 | 0.999 | <0.001 |
| **7** | 74.41 | 1097 | 0.979 | <0.001 | 25.62 | 2120 | 0.883 | <0.001 | 52.05 | 1390 | 0.989 | <0.001 |
| **8** | 103.4 | 1155 | 0.994 | <0.001 | 21.54 | 1369 | 0.813 | 0.002 | 67.79 | 1144 | 0.997 | <0.001 |
| **9** | 86.47 | 2368 | 0.982 | <0.001 | 40.83 | 1456 | 0.986 | <0.001 | 45.75 | 1637 | 0.997 | <0.001 |
| **10** | 81.67 | 961.4 | 0.996 | <0.001 | 24.34 | 1486 | 0.938 | <0.001 | 58.04 | 875.9 | 0.994 | <0.001 |
| **11** | 88.77 | 1175 | 0.989 | <0.001 | 24.98 | 1668 | 0.836 | 0.001 | 39.71 | 1086 | 0.979 | <0.001 |
| **12** | 76.40 | 1156 | 0.998 | <0.001 | 24.13 | 1917 | 0.874 | <0.001 | 61.95 | 1210 | 0.996 | <0.001 |
| **13** | 46.55 | 2856 | 0.935 | <0.001 | 36.04 | 1829 | 0.990 | <0.001 | 61.15 | 2107 | 0.977 | <0.001 |
| **14** | 98.46 | 1727 | 0.998 | <0.001 | 30.48 | 2321 | 0.932 | <0.001 | 58.13 | 1318 | 0.997 | <0.001 |
| **15** | 72.38 | 1614 | 0.996 | <0.001 | 66.18 | 2120 | 0.987 | <0.001 | 53.59 | 1623 | 0.996 | <0.001 |
| **16** | 84.32 | 1135 | 0.995 | <0.001 | 13.15 | 1449 | 0.452 | 0.068 | 60.78 | 1312 | 0.997 | <0.001 |
| **17** | 59.14 | 2652 | 0.984 | <0.001 | 11.00 | 1115 | 0.345 | 0.126 | 33.33 | 1353 | 0.980 | <0.001 |
| **18** | 114.8 | 1665 | 0.991 | <0.001 | 19.11 | 1899 | 0.693 | 0.01 | 50.14 | 938.5 | 0.992 | <0.001 |
| **19** | 43.64 | 829.9 | 0.998 | <0.001 | 17.87 | 1552 | 0.801 | 0.003 | 43.49 | 1022 | 0.986 | <0.001 |
| **20** | 67.51 | 1039 | 0.995 | <0.001 | 8.137 | 1504 | 0.341 | 0.129 | 47.56 | 932.2 | 0.997 | <0.001 |
| **21** | 69.70 | 2184 | 0.993 | <0.001 | 8.478 | 1115 | 0.466 | 0.062 | 21.98 | 1372 | 0.793 | 0.003 |
| **22** | 69.56 | 1267 | 0.993 | <0.001 | 35.52 | 1746 | 0.977 | <0.001 | 44.08 | 849.6 | 0.997 | <0.001 |
| **23** | 55.40 | 921.7 | 0.997 | <0.001 | -1.769 | 987.8 | 0.008 | 0.828 | 41.68 | 1053 | 0.994 | <0.001 |
| **24** | 56.73 | 850.3 | 0.990 | <0.001 | 7.109 | 1006 | 0.387 | 0.1 | 46.91 | 914.6 | 0.996 | <0.001 |

**Supplementary Information 2**: Loss stiffness (Eq. 8) regression analyses of the osteochondral core and its constituents. Stiffness coefficients (A_L_) and constants (B_L_) are in N/mm. p < 0.05 indicates that the logarithmic regression analysis was significant.

|  | **Cartilage k’’** | | | | **Bone k’’** | | | | **Core k’’** | | | |
| --- | --- | --- | --- | --- | --- | --- | --- | --- | --- | --- | --- | --- |
|  | **A_L_** | **B_L_** | **r^2^** | **p** | **A_L_** | **B_L_** | **r^2^** | **p** | **A_L_** | **B_L_** | **r^2^** | **P** |
| **1** | 13.36 | 289.7 | 0.838 | 0.001 | -10.03 | 104.9 | 0.845 | 0.001 | -5.159 | 120.4 | 0.727 | 0.007 |
| **2** | -5.872 | 272.8 | 0.566 | 0.031 | -6.976 | 91.88 | 0.819 | 0.002 | -2.152 | 99.78 | 0.481 | 0.056 |
| **3** | 12.87 | 185.6 | 0.903 | <0.001 | -5.699 | 82.75 | 0.780 | 0.004 | -1.809 | 94.50 | 0.205 | 0.26 |
| **4** | 3.800 | 195.3 | 0.412 | 0.086 | -7.099 | 116.6 | 0.866 | <0.001 | 0.638 | 79.17 | 0.076 | 0.509 |
| **5** | -5.509 | 204.9 | 0.483 | 0.056 | -8.119 | 112.1 | 0.711 | 0.009 | -3.411 | 118.3 | 0.670 | 0.013 |
| **6** | 0.003 | 213.4 | 0.000 | 0.998 | -3.118 | 82.50 | 0.405 | 0.09 | -0.521 | 130.6 | 0.057 | 0.571 |
| **7** | 17.89 | 192.2 | 0.959 | <0.001 | -9.013 | 106.7 | 0.849 | 0.001 | -1.967 | 96.17 | 0.283 | 0.175 |
| **8** | 14.93 | 242.0 | 0.915 | <0.001 | -6.147 | 85.67 | 0.935 | <0.001 | -2.938 | 134.7 | 0.595 | 0.025 |
| **9** | -10.91 | 137.0 | 0.725 | 0.007 | -8.639 | 115.9 | 0.842 | 0.001 | -6.000 | 103.1 | 0.852 | 0.001 |
| **10** | 5.621 | 225.1 | 0.633 | 0.018 | -7.196 | 88.62 | 0.920 | <0.001 | 2.146 | 113.4 | 0.469 | 0.061 |
| **11** | 8.202 | 200.0 | 0.854 | 0.001 | -5.584 | 87.29 | 0.710 | 0.009 | -1.402 | 83.99 | 0.134 | 0.372 |
| **12** | 10.49 | 208.4 | 0.809 | 0.002 | -6.784 | 103.1 | 0.674 | 0.013 | -1.463 | 134.8 | 0.212 | 0.25 |
| **13** | -2.726 | 177.5 | 0.040 | 0.635 | -5.109 | 113.2 | 0.767 | 0.004 | -10.30 | 135.3 | 0.694 | 0.01 |
| **14** | -2.989 | 261.3 | 0.227 | 0.233 | -10.35 | 114.4 | 0.723 | 0.007 | -1.000 | 112.7 | 0.177 | 0.299 |
| **15** | 8.461 | 170.3 | 0.896 | <0.001 | -12.22 | 140.0 | 0.800 | 0.003 | -2.764 | 103.5 | 0.440 | 0.073 |
| **16** | 4.711 | 220.8 | 0.718 | 0.008 | -5.043 | 80.35 | 0.596 | 0.025 | -1.391 | 109.1 | 0.185 | 0.288 |
| **17** | -8.352 | 165.3 | 0.903 | 0.887 | -7.508 | 81.02 | 0.959 | <0.001 | -5.593 | 89.94 | 0.857 | <0.001 |
| **18** | -8.990 | 283.8 | 0.690 | 0.011 | -5.965 | 87.14 | 0.686 | 0.011 | 0.860 | 80.84 | 0.135 | 0.371 |
| **19** | 11.02 | 130.5 | 0.914 | <0.001 | -4.184 | 88.58 | 0.644 | 0.017 | -0.398 | 78.99 | 0.082 | 0.493 |
| **20** | 15.92 | 173.5 | 0.949 | <0.001 | -5.411 | 94.84 | 0.835 | 0.002 | 0.513 | 91.56 | 0.126 | 0.389 |
| **21** | -8.061 | 149.6 | 0.828 | 0.002 | -3.858 | 60.48 | 0.848 | 0.001 | -2.987 | 80.58 | 0.698 | 0.01 |
| **22** | -3.605 | 190.7 | 0.407 | 0.089 | -7.176 | 118.3 | 0.746 | 0.006 | 1.467 | 79.63 | 0.358 | 0.117 |
| **23** | 5.189 | 148.5 | 0.762 | 0.005 | -2.455 | 49.26 | 0.346 | 0.125 | -2.190 | 91.56 | 0.426 | 0.079 |
| **24** | 10.76 | 150.3 | 0.919 | <0.001 | -2.429 | 64.45 | 0.788 | 0.003 | -0.200 | 101.7 | 0.011 | 0.808 |

**Supplementary Information 3**: Storage modulus (Eq. 9) and loss modulus (Eq. 10) regression analyses of the osteochondral core and its constituents. Moduli coefficients (C and C_L_) and constants (D and D_L_) are in MPa*.* *p* < 0.05 indicates that the logarithmic regression analysis was significant.

|  | **Cartilage E’** | | | | **Bone E’** | | | | **Cartilage E’’** | | | | **Bone E’’** | | | |
| --- | --- | --- | --- | --- | --- | --- | --- | --- | --- | --- | --- | --- | --- | --- | --- | --- |
|  | **C** | **D** | **r^2^** | **p** | **C** | **D** | **r^2^** | **P** | **C_L_** | **D_L_** | **r^2^** | **p** | **C_L_** | **D_L_** | **r^2^** | **p** |
| **1** | 2.4 | 29.9 | 0.98 | <0.001 | 1.7 | 113.5 | 0.89 | <0.001 | 0.3 | 5.9 | 0.84 | 0.001 | -0.6 | 6.5 | 0.85 | 0.001 |
| **2** | 4.4 | 63.0 | 1.00 | <0.001 | 0.7 | 97.8 | 0.20 | 0.27 | -0.2 | 11.2 | 0.57 | 0.031 | -0.5 | 6.0 | 0.82 | 0.002 |
| **3** | 1.5 | 30.3 | 0.99 | <0.001 | 1.3 | 98.0 | 0.91 | <0.001 | 0.2 | 3.2 | 0.90 | <0.001 | -0.4 | 5.5 | 0.78 | 0.004 |
| **4** | 2.6 | 35.7 | 0.99 | <0.001 | 2.7 | 135.9 | 0.93 | <0.001 | 0.1 | 5.6 | 0.41 | 0.086 | -0.6 | 9.4 | 0.87 | <0.001 |
| **5** | 1.5 | 35.5 | 0.99 | <0.001 | 0.9 | 89.1 | 0.41 | 0.088 | -0.1 | 3.7 | 0.48 | 0.056 | -0.5 | 6.4 | 0.71 | 0.009 |
| **6** | 2.2 | 34.6 | 1.00 | <0.001 | 1.4 | 102.4 | 0.82 | 0.002 | 0.0 | 6.9 | 0.00 | 0.998 | -0.2 | 5.6 | 0.41 | 0.09 |
| **7** | 2.0 | 29.0 | 0.98 | <0.001 | 2.1 | 177.2 | 0.88 | <0.001 | 0.5 | 5.1 | 0.96 | <0.001 | -0.8 | 8.9 | 0.85 | 0.001 |
| **8** | 2.6 | 29.5 | 0.99 | <0.001 | 1.6 | 99.9 | 0.81 | 0.002 | 0.4 | 6.2 | 0.92 | <0.001 | -0.4 | 6.3 | 0.94 | <0.001 |
| **9** | 1.4 | 37.5 | 0.98 | <0.001 | 2.7 | 97.5 | 0.99 | <0.001 | 0.0 | 2.8 | 0.04 | 0.635 | -0.6 | 7.8 | 0.84 | 0.001 |
| **10** | 2.7 | 31.2 | 1.00 | <0.001 | 1.3 | 81.8 | 0.94 | <0.001 | 0.2 | 7.3 | 0.63 | 0.018 | -0.4 | 4.9 | 0.92 | <0.001 |
| **11** | 2.6 | 34.3 | 0.99 | <0.001 | 1.7 | 113.8 | 0.84 | <0.001 | 0.2 | 5.8 | 0.85 | <0.001 | -0.4 | 6.0 | 0.71 | 0.009 |
| **12** | 2.0 | 30.8 | 1.00 | <0.001 | 1.4 | 110.6 | 0.87 | <0.001 | 0.3 | 5.6 | 0.81 | 0.002 | -0.4 | 5.9 | 0.67 | 0.013 |
| **13** | 0.6 | 36.8 | 0.94 | <0.001 | 2.5 | 124.8 | 0.99 | <0.001 | -0.1 | 1.8 | 0.73 | 0.007 | -0.3 | 7.7 | 0.77 | 0.004 |
| **14** | 2.4 | 42.9 | 1.00 | <0.001 | 2.2 | 164.7 | 0.93 | <0.001 | -0.1 | 6.5 | 0.23 | 0.233 | -0.7 | 8.1 | 0.72 | 0.007 |
| **15** | 1.6 | 36.7 | 1.00 | <0.001 | 4.6 | 146.2 | 0.99 | <0.001 | 0.2 | 3.9 | 0.90 | <0.001 | -0.8 | 9.7 | 0.80 | 0.003 |
| **16** | 2.3 | 30.3 | 1.00 | <0.001 | 0.7 | 80.7 | 0.45 | 0.068 | 0.1 | 5.9 | 0.72 | 0.008 | -0.3 | 4.5 | 0.60 | 0.025 |
| **17** | 1.1 | 49.4 | 0.98 | <0.001 | 0.7 | 68.8 | 0.35 | 0.126 | -0.2 | 3.1 | 0.90 | <0.001 | -0.5 | 5.0 | 0.96 | <0.001 |
| **18** | 3.8 | 55.3 | 0.99 | <0.001 | 1.1 | 109.6 | 0.69 | 0.01 | -0.3 | 9.4 | 0.69 | 0.011 | -0.3 | 5.0 | 0.69 | 0.011 |
| **19** | 1.5 | 29.1 | 1.00 | <0.001 | 1.0 | 89.5 | 0.80 | 0.003 | 0.4 | 4.6 | 0.91 | <0.001 | -0.2 | 5.1 | 0.54 | 0.017 |
| **20** | 2.1 | 32.7 | 1.00 | <0.001 | 0.5 | 89.8 | 0.34 | 0.129 | 0.5 | 5.5 | 0.50 | <0.001 | -0.3 | 5.7 | 0.84 | 0.002 |
| **21** | 0.9 | 29.3 | 0.99 | <0.001 | 0.5 | 71.0 | 0.47 | 0.062 | -0.1 | 2.0 | 0.83 | 0.002 | -0.2 | 3.9 | 0.85 | 0.001 |
| **22** | 2.5 | 45.1 | 0.99 | <0.001 | 2.0 | 97.3 | 0.98 | <0.001 | -0.1 | 6.8 | 0.41 | 0.089 | -0.4 | 6.6 | 0.75 | 0.006 |
| **23** | 1.7 | 28.1 | 1.00 | <0.001 | -0.1 | 68.0 | 0.01 | 0.828 | 0.2 | 4.5 | 0.76 | 0.005 | -0.2 | 3.4 | 0.35 | 0.125 |
| **24** | 1.6 | 23.7 | 0.99 | <0.001 | 0.5 | 68.1 | 0.39 | 0.1 | 0.3 | 4.2 | 0.92 | <0.001 | -0.2 | 4.4 | 0.79 | 0.003 |
